# The Role of Artificial Intelligence Model Documentation in Translational Science: Scoping Review

**DOI:** 10.2196/45903

**Published:** 2023-07-14

**Authors:** Tracey A Brereton, Momin M Malik, Mark Lifson, Jason D Greenwood, Kevin J Peterson, Shauna M Overgaard

**Affiliations:** 1 Center for Digital Health Mayo Clinic Rochester, MN United States; 2 Department of Family Medicine Mayo Clinic Rochester, MN United States

**Keywords:** health, informatics, artificial intelligence, machine learning, documentation, explainability, ethics, translational science, scoping review, medical modeling software, clinical decision support, decision support intervention

## Abstract

**Background:**

Despite the touted potential of artificial intelligence (AI) and machine learning (ML) to revolutionize health care, clinical decision support tools, herein referred to as medical modeling software (MMS), have yet to realize the anticipated benefits. One proposed obstacle is the acknowledged gaps in AI translation. These gaps stem partly from the fragmentation of processes and resources to support MMS transparent documentation. Consequently, the absence of transparent reporting hinders the provision of evidence to support the implementation of MMS in clinical practice, thereby serving as a substantial barrier to the successful translation of software from research settings to clinical practice.

**Objective:**

This study aimed to scope the current landscape of AI- and ML-based MMS documentation practices and elucidate the function of documentation in facilitating the translation of ethical and explainable MMS into clinical workflows.

**Methods:**

A scoping review was conducted in accordance with PRISMA-ScR (Preferred Reporting Items for Systematic Reviews and Meta-Analyses extension for Scoping Reviews) guidelines. PubMed was searched using Medical Subject Headings key concepts of *AI, ML*, *ethical considerations*, and *explainability* to identify publications detailing AI- and ML-based MMS documentation, in addition to snowball sampling of selected reference lists. To include the possibility of implicit documentation practices not explicitly labeled as such, we did not use *documentation* as a key concept but as an inclusion criterion. A 2-stage screening process (title and abstract screening and full-text review) was conducted by 1 author. A data extraction template was used to record publication-related information; barriers to developing ethical and explainable MMS; available standards, regulations, frameworks, or governance strategies related to documentation; and recommendations for documentation for papers that met the inclusion criteria.

**Results:**

Of the 115 papers retrieved, 21 (18.3%) papers met the requirements for inclusion. Ethics and explainability were investigated in the context of AI- and ML-based MMS documentation and translation. Data detailing the current state and challenges and recommendations for future studies were synthesized. Notable themes defining the current state and challenges that required thorough review included bias, accountability, governance, and explainability. Recommendations identified in the literature to address present barriers call for a proactive evaluation of MMS, multidisciplinary collaboration, adherence to investigation and validation protocols, transparency and traceability requirements, and guiding standards and frameworks that enhance documentation efforts and support the translation of AI- and ML-based MMS.

**Conclusions:**

Resolving barriers to translation is critical for MMS to deliver on expectations, including those barriers identified in this scoping review related to bias, accountability, governance, and explainability. Our findings suggest that transparent strategic documentation, aligning translational science and regulatory science, will support the translation of MMS by coordinating communication and reporting and reducing translational barriers, thereby furthering the adoption of MMS.

## Introduction

### Background

Artificial intelligence (AI)- and machine learning (ML)-based tools have been hailed as having the potential to revolutionize health care with innovative, efficient, and intuitive approaches to care [1-4]. The successful integration of such tools into clinical settings necessitates a meticulous evaluation conducted by interdisciplinary teams throughout the AI life cycle, ensuring favorable outcomes; however, the promised value of delivering scalable and sustained value for patients has yet to be realized, as the field has recognized a gap in implementation [1,3-7]. *Clinical decision support* (CDS) tools range from computerized alerts and reminders, clinical guidelines, order sets, patient data reports and summaries, documentation templates, diagnostic support, among others, and aim to provide clinicians, staff, and patients with knowledge and person-specific information to support and enhance decision-making in the clinical workflow [8]. The Office of the National Coordinator for Health Information Technology has proposed revising and renaming the CDS criterion in the 2023 Health Data, Technology, and Interoperability: Certification Program Updates, Algorithm Transparency, and Information Sharing Proposed Rule to reflect the array of contemporary and emerging functionalities, data elements, and software applications that aid decision-making in health care and introducing *decision support interventions* [9]. Decision support intervention encompasses “technology that is intended to support decision-making based on algorithms or models that derive relationships from training or example data and then are used to produce an output or outputs related to, but not limited to, prediction, classification, recommendation, evaluation, or analysis” [9]. In this paper, we refer to such tools broadly as *AI- and ML-based medical modeling software* (MMS), as our core concern is with models in medical care based on the AI and ML approach of algorithmic modeling [5] that finds optimal (often noncausal) correlations, rather than traditional statistical theory–based models that try to capture causal processes and underlying mechanisms. This style of modeling introduces novel questions around validation, methodology, communication, coordination, and ethics. We introduce a new term to focus on these specific issues because, although AI- and ML-based MMS may be used for CDS or be implemented in software as a medical device (SaMD), CDS includes systems that are not AI- and ML-based and the software in SaMD may not involve any AI or ML modeling component.

Although progress has been made in AI and ML innovation and many solutions are being developed with high-performance metrics, most software remains within the realm of research rather than real-world settings, and even the most technology-literate academic institutions are not routinely using AI and ML in clinical workflows [1-3,6]. Seneviratne et al [2] asks, “If model performance is so promising, why is there such a chasm between development and deployment?” To recognize the importance of accounting for the complexities of health care delivery throughout the life cycle of MMS production, it is essential to understand what barriers exist and then work to close the implementation gap.

Documentation of MMS may reduce these barriers, but it must first go beyond the assessment of technical performance and involve a holistic, interdisciplinary evaluation process that complements and works in tandem with the software life cycle [1,3,4,7]. Currently, the available documentation frameworks for MMS are fragmented, and there needs to be more guidance spanning all disciplines and stages of development [1-3,7]. Li et al [1] call on the need for a “delivery science” that encompasses a broad set of tools to encourage iterative design thinking among data scientists and clinical informaticists and to promote implementation science techniques across health care operations, ethics, and so on that can be transparently documented. Similarly, the International Telecommunications Union and World Health Organization (WHO) Focus Group on Artificial Intelligence for Health [10] calls for the alignment of 4 pillars—ethics, regulations, technology, and clinical evaluation and use cases—to appropriately evaluate and guide development and ensure the feasibility of a solution to generate sufficient knowledge and evidence to support implementation.

The lack of available, professionally accepted, and ubiquitous references describing appropriate documentation makes it challenging to create evidence supporting the safe and effective translation of MMS from research into clinical practice. To help close the gap, comprehensive and practical documentation processes must be in place to capture critical information about software, incorporating all phases of the software life cycle.

### Previous Studies

An initial literature review was conducted to understand the current state of documentation and its impact on the translation of AI- and ML-based MMS into clinical practice. Papers obtained through keyword searches in PubMed were analyzed and synthesized. The search focused on characterizing the extent of existing materials rather than exploring any potential issues that may have been overlooked. We found no consensus on “best practices” for documentation around what we identified as AI- and ML-based MMS. However, we recorded the relevant reporting guidelines offered by government and oversight bodies, ethical principles, and theoretical guiding frameworks. Overlapping principles prioritized explainability, transparency, accountability, and trustworthiness, but descriptions were highly variable throughout the field [11-13]. Despite its potential, the adoption of AI- and ML-based MMS remained fragmented, and there were reports about bias after deployment that put patient safety at risk, providing inaccurate or skewed outcomes and recommendations, propagating inequalities, and introducing group harm [14-17]. The findings were presented to multidisciplinary stakeholders across the MMS life cycle. This workshop highlighted the relevance and urgency for continuing studies regarding the current state and documentation challenges to support the development and translation of AI- and ML-based MMS.

### Objectives

The findings from the initial literature review and internal research motivated this scoping review to further evaluate the current state and direction of AI- and ML-based MMS documentation. This study aimed to scope AI- and ML-based MMS documentation practices and define the role of documentation in facilitating the translation of ethical and explainable MMS into clinical workflows.

## Methods

### Study Design

Covidence (Veritas Health Innovation) [18], a web-based collaboration software platform that streamlines the production of systematic and other literature reviews and developed in accordance with PRISMA-ScR (Preferred Reporting Items for Systematic Reviews and Meta-Analyses extension for Scoping Reviews) guidelines [19], was leveraged to ensure compliance with scoping review standards and facilitate a systematic process to define eligibility criteria, search the literature, screen results, select evidence for inclusion, and conduct data extraction.

### Define the Eligibility Criteria

Key concepts were identified as *AI, ML*, *ethical considerations*, and *explainability* to broadly search the literature for evidence of recommendations to support AI- and ML-based MMS documentation practices, scope the development of ethical and explainable software, and define the role of documentation for safe and ethical translation. The use of broad concepts aimed to account for the range of AI- and ML-based MMS and the available recommendations for documentation practices. To include the possibility of implicit documentation practices not explicitly labeled as such, we did not use *documentation* as a key concept but as an inclusion criterion. Keywords and Medical Subject Heading terms were used to support and generate the search query and search constraints including literature found only in the PubMed database; publications dated after 2015; and journals identified to be relevant to the study objectives as defined by the “find journals” functionality of the website, Jane [20]—a website that mines documents in PubMed to find the best matching journals, authors, or papers. The inclusion and exclusion criteria used to assess the publications retrieved are provided in Textbox 1. The search query used for this scoping review, combined using the AND query function, is available in Multimedia Appendix 1.

**Inclusion and exclusion criteria that were used to assess the publications retrieved in the scoping review.**

**Inclusion criteria**

- Paper type—all study designs and publication types
- Language—English
- Setting—artificial intelligence (AI) and machine learning (ML) in health care
- Topic—documentation practices involving AI, ML, ethics, and explainability

**Exclusion criteria**

- Paper type—none
- Language—non-English
- Setting—non–health care
- Topic—topics other than documentation practices involving AI, ML, ethics, and explainability

### Searching and Screening the Literature

Publications were retrieved and imported into Covidence to conduct a 2-stage screening process, including title and abstract screening, followed by a full-text review completed by 1 author. Because this was a scoping review with a relatively small sample size, it was determined that additional coders were not needed. Papers that did not meet the predefined eligibility criteria and those that were not health care related or did not evaluate the current state and direction of AI- and ML-based MMS documentation and translation were excluded.

### Data Extraction

Included papers were analyzed to consolidate evidence of current documentation practices, scope the development of ethical and explainable software, and define the role of documentation in facilitating translation. A data extraction template (Multimedia Appendix 2) was developed and used by 1 author within Covidence to determine the extraction criteria and synthesize the data of the included papers in a consistent format. To effectively consolidate the evidence of the current state and challenges to support the objectives of this study, literature was synthesized by *bias, accountability, governance*, *explainability*, and *detailed communicated recommendations.*

## Results

### Characteristics of Included Literature

**Figure 1:**
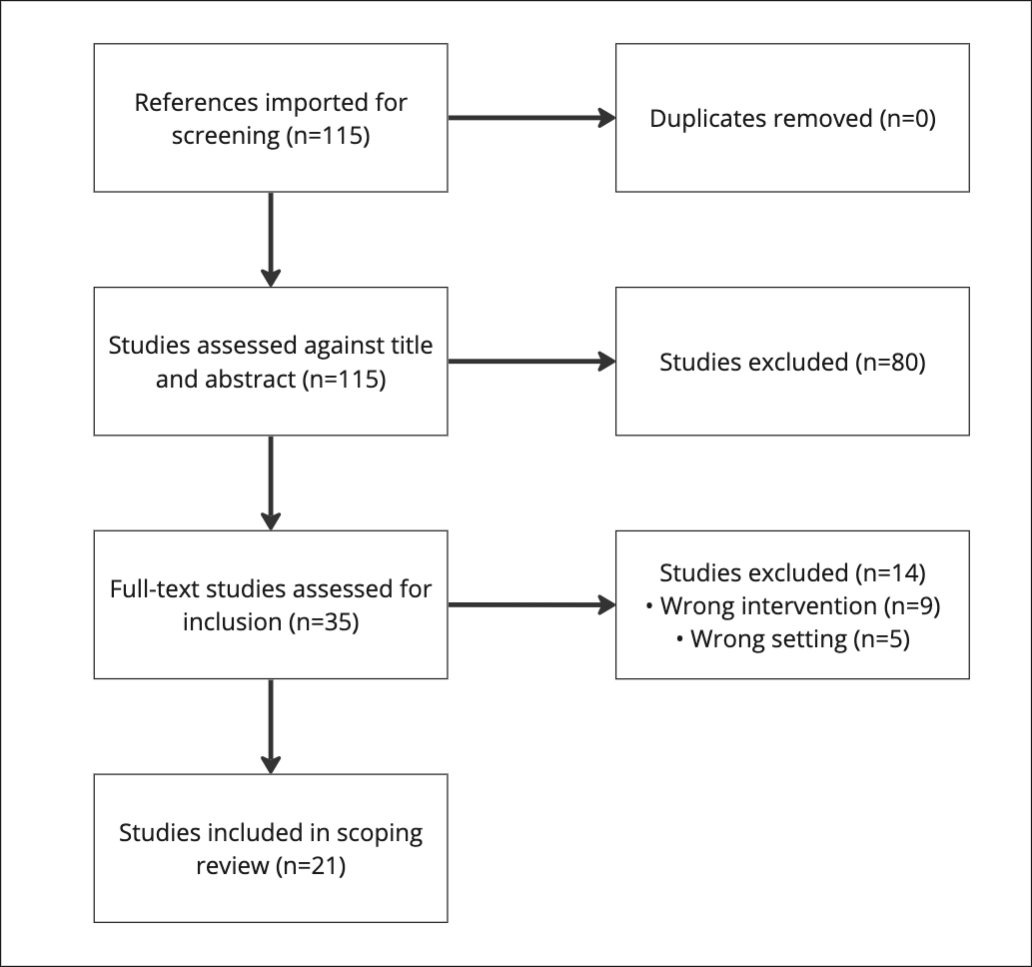
PRISMA-ScR (Preferred Reporting Items for Systematic Reviews and Meta-Analyses extension for Scoping Reviews) flow diagram summarizing the screening process used to conduct the scoping review.

The initial search in PubMed retrieved 115 papers, which were imported into Covidence for screening and extraction, as shown in the PRISMA-ScR guidelines flow diagram (Figure 1). The PRISMA-ScR checklist is available in Multimedia Appendix 3. Title and abstract screening were conducted, and 29.6% (34/115) of the papers met the eligibility criteria for inclusion. After title and abstract screening, papers were subjected to full-text reviews, where 62% (21/34) of them met the eligibility criteria for inclusion. Of the 21 papers, all (n=21, 100%) included *AI*, *ML*, or *CDS* as keywords, 15 (71%) included *ethics* or *bias* as keywords, and 12 (57%) had *explainability*, *interpretability*, *translation*, *governance*, or *policymaking* as keywords. Relevance was of importance because the current state was being evaluated. Therefore, an analysis of publication year was conducted; of the 21 papers, 3 (14%) were published in 2019, a total of 9 (43%) in 2020, a total of 7 (33%) in 2021, and 2 (10%) in 2022. Additional characteristics of the publications included are available in Multimedia Appendix 4 [13,21-40].

Snowball sampling [41] was used to collect materials such as documents from governmental and regulatory agencies not indexed in PubMed and those referenced in the retrieved documents but not explained with sufficient detail or explained with minimal context. This allowed a more extensive summary of existing frameworks, principles, and standards for ethics (Multimedia Appendix 5 [22-26,28,29,39,40]) and explainability (Multimedia Appendix 6 [22-24,27,28,31,32,34,36-39]) on which existing literature relies on but falls outside of peer-reviewed, indexed literature.

### Current State and Challenges

#### Overview

Existing studies focus on how the translation of AI- and ML-based MMS into patient care settings requires collaboration throughout the AI life cycle and across interdisciplinary specialties of a product team. Furthermore, effective communication and documentation strategies enable collaborative teams to (1) report about developments, (2) inform system progression, and (3) ensure the cross-functional evaluation and documentation of functions from system functionality to ethical considerations. Product teams face challenges involving insufficient collaboration, isolated and fragmented development and documentation processes that fail to prioritize knowledge continuity, timing of interventions, and deficient or unavailable reporting resources needed to progress production, thus disrupting translation into clinical practice [21-23].

The absence of practical tools and best practices to guide translational approaches throughout the AI life cycle contributed to the previously discussed implementation gap. Guidance for ethics and explainability is expected to meet the needs of a broad range of stakeholders, but what constitutes AI ethics and explainability best practices, who determines them, how principles are applied and regulated in practice, and how it is documented still needs to be defined [13,21,22,24,25,42]. Reddy et al [25] noted that “there is little dialogue or recommendations as to how to practically address these concerns in healthcare,” suggesting the need for standardized or mandated regulations to guide processes of collaboration, documentation, and compliance and enhance translational processes with sufficient evidence to support production [22].

Ethics and explainability were investigated in the context of AI- and ML-based MMS documentation and translation. Current challenges involving bias, accountability, governance, and explainability were notable themes requiring thorough review as they pose present barriers to AI- and ML-based MMS translation.

#### Bias

Differential model performance is a limiting factor in the applicability and deployment of AI- and ML-based MMS owing to the direct implications that biased models have on equity, feasibility of use cases, patient safety, performance, and time and cost of corrections [22,23,26-28]. There is a lack of resources to guide what to do when models perform systematically less well on identifiable patient subgroups and specifically marginalized subgroups, which are often found to be undetected at deployment and identified in ex post reviews [22,23,26,27]. Reddy et al [25] used the adage “biases in, biases out” to describe the influence of bias and how algorithmic decisions and output directly reflect input [25,29]. When efforts are not taken to increase data diversity and quality, then the existing digital divide, social inequalities, and health care disparities are widened; underrepresented and at-risk populations are penalized; vulnerabilities are exacerbated; and harm is increased [28,30,31]. Fragmented processes from which MMS are derived, often from single institutions with single-institution data for training, testing, and validation, may incorrectly assume that the target population mimics the training data population (demographically, socioeconomically, medically, etc). This, in turn, limits external reproducibility owing to generalizability concerns across fields [24]. If created from single-institution data and not externally validated, risks extend beyond appropriate use cases, further jeopardizing patient safety and providing insufficient outputs [24,25,28,31,32]. Kerasidou [33] pointed to the regularity with which inequitable models are deployed, emphasizing the need for evidence regarding prospective evaluation and representativeness relative to training and deployment environments to minimize consequences of patient harm and increase model performance, accuracy, fairness, and trust [30].

#### Accountability

The introduction of AI solutions into clinical workflows has raised questions about accountability. Does a clinician’s responsibility extend to decisions made from following model outputs, including any harmful or poor outcomes [22,28,33,34]? Must clinicians disclose the level of autonomy or obtain consent from patients regarding the use of MMS in their care [21,25,28,29]? If a clinician’s decision contradicts the AI output, how must a clinician proceed, and who is liable [23,25,32,34]? Navigating accountability about the intent of MMS requires the constructs of safety, responsibility, autonomy, consent, and trust to be deeply considered throughout the AI life cycle [23].

With the application and expectation of MMS aiming to enhance clinician decisions and patient outcomes, proactive solutions to navigate responsibility in response to incorrectness or harm are also imperative. Although the level of autonomy upon which MMS operates varies, when systems fail, there is an expectation of accountability [25,28]. Contradicting opinions between clinicians and tools present unique problems. There exist different approaches to defensive medicine within AI. For instance, when a clinician and model have opposing diagnostic decisions, some clinicians are compelled to follow their instinct and practical experience. In contrast, others are obliged to defer to a model’s logic. Regardless of clinical outcome, patients often remain uninformed as to whether the final decision heeds or disregards an AI recommendation [21].

Responsibility and liability were associated with the usability and trust of MMS within patient-clinician relationships and public support for the integration of the MMS [23,25,30,33]. Concerns surrounding the dehumanization of health care are often present when MMS is deployed, such as the risk of clinician overreliance, disruptions in patient-clinician relationships, and management of false societal expectations of AI [21,28,33]. Overreliance reduces or eliminates patient encounters with clinicians, changing the landscape of patient involvement and potentially missing data that would otherwise be collected outside electronic health records [21,25]. Grote and Berens [21] stated that “facilitating successful collaboration between ML algorithms and clinicians is proving to be a recalcitrant problem that may exacerbate ethical issues in clinical medicine.”

#### Governance

A gap between ethics and regulation is said to exist with the range of AI- and ML-based MMS classifications [25,30]. For the comparable category of medical device software, there are many international standards and guidance documents for building high-quality, safe, and effective medical device software, but there is no explicit definition of ethical criteria; however, the impact of the regulatory process accounts for many of the identified ethical considerations. Various ideologies, principles, and frameworks try to define what ethical AI is, including the AI Ethics Principles, Ethically Aligned Design principles developed by the Institute of Electrical and Electronics Engineers (IEEE) and Trustworthy AI principles defined by the High-level Expert Group on Artificial Intelligence, but best practices are found to be scattered [26,32,38]. Software developers desire increased guidance, resources, and reporting guidelines to develop and deploy ethically designed MMS fields [22,34]. Clinicians, one of the primary end users of MMS, communicate the need for documentation resources to serve as training material for the intended use and a means of communicating the objective, functionality, and limitations of MMS to ensure the appropriate and actionable translation of software into practice [21,22]. In addition to ethical considerations, although adequate information or recommendations, privacy protection, data integrity, and regulatory frameworks may be facilitated by government oversight, evidence suggests that AI- and ML-based MMS are not deployed with sufficient levels of documentation to support explainability [30]. With such leadership and resources allocated to MMS, institutions can avoid inconclusive, inscrutable, and misguided evidence; unfair outcomes; and the lack of traceability and transformative effectiveness [32,34]. Subjecting researchers to self-monitoring of ethical conduct and determining the level of explainability to accompany MMS is of substantial concern but may be combated with increased reporting rigor and guardrails for evaluation and reporting of evidence [13,22,25,26,30].

Although regulators guide how to deploy safe and effective medical device software, including requiring robust clinical validations and postdeployment monitoring and surveillance programs, these processes may be out of the scope of requirements for some AI- and ML-based MMS, including those with limited or no regulation requirements by the Food and Drug Administration (FDA). Questions such as “How much accuracy [or other relevant performance metric] is sufficient for deployment?” “What level of transparency is required?” and “Do we understand when the model outputs are likely to be unreliable and therefore should not be trusted?” have made defining expectations and requirements for governance difficult, especially for varying levels of system autonomy and risk [13,21,24,25,35]. Although frameworks and principles have been developed by various organizations with goals of increasing explainability (FDA; European Union General Data Protection Regulation; WHO; IEEE; and Findability, Accessibility, Interoperability, and Reusability principles), no recognition of established best practices were identified, only recommended principles to abide by, such as those defined within the AI Ethics Principles [23,24,31,32,35,36]. Reported consequences of fragmented documentation resources and reporting requirements include the lack of explainability, accountability, validation, transparency, and trust [22,25,28,29].

#### Explainability

Although the field points to explainability to help facilitate successful documentation and translation, the literature acknowledges difficulties with such resources and best practices that are expected to contribute to achieving explainable AI- and ML-based MMS [13,22,36,37]. Although some have argued for distinguishing interpretability (inherently interpretable models) from explainability (post hoc, simplified summaries of model functionality that supplement the model) [43], within the scoped literature, the terms interpretation or interpretability and explanation or explainability were used interchangeably. Since the overall use is closer to the proposed meaning of *explainability*, we exclusively use this term. Explainability, then, defined as understandable (post hoc and supplementary) explanations of ML model outcomes, accounts for how users should be able to understand the logic of ML modeling to implement it appropriately within clinical workflows [13,27,38]. Establishing explainability is functionally complex without supplemental documentation, creating challenges in understanding decision logic, deploying transparent tools, and defining accountability and responsibility (especially with requirements varying with different risk classifications) and threatening patient safety and trust [13,21,25,27,37].

The complex nature of AI- and ML-based MMS, comprising multifaceted computations that drive decision-aiding output, complicates explainability and initiates debate regarding the prioritization of performance versus explainability in system development [13,25]. “Black boxes” have been found to make the clinical application and decision procedures “notoriously hard to interpret and explain in detail,” limiting the ability to identify and document technical and logical justifications for decisions and conflicting with core values of patient consent and awareness about the role AI in their care [13,25,32,36,38]. Kerasidou [33] stated that with black box systems, “the ‘thinking process’ by which outcomes are produced is not obvious to those who use the AI or even to those who develop it,” raising explainability, transparency, and justification concerns from developers to clinicians and from clinicians to patients [13,32,44]. Amann et al [13] questioned whether, owing to their complexity, black boxes are even documentable. Without a way to document and report about explanations of AI, it is “hard to determine if differences in diagnoses reflect diagnostically relevant differences between patients or if they are instances of bias or diagnostic errors and over-/underdiagnosis,” further emphasizing the need to mitigate and address biases before deployment [36]. Once clinicians can no longer comprehend decisions fully, they cannot explain to the patient how specific outcomes or recommendations were derived, thus affecting patient safety, trust, and care plans [13,27]. The literature describes the necessity to interpret MMS logic because omitting it has been found to “pose a threat to core ethical values in medicine and may have detrimental consequences for individual and public health,” including evidence of disregard for ethical and regulatory practices, unsuitable clinical application, and making it impossible to investigate and rectify causes of errors; however, Yoon et al [27] argued that the extent to which explainability is required still needs to be determined [13,27,33,38].

Beyond an understanding of system complexity, explanations of system functionality were found to be critical for developers, stakeholders, clinicians, and patients to understand a system about clinical applicability and intended use, despite varying levels of familiarity with AI [38,39]. The context of appropriate clinical application is critical to disclose, owing to the impact of training data on system performance and deployment environment, but developing explainable models that satisfy requirements of providing supporting information for clinical decision-making proves to be challenging, given that clinical decisions are made based on different modalities and reasoning strategies [21,22,35]. When explanations detect, analyze, and assess artifacts of MMS throughout design, development, and implementation, the field may anticipate more informed and trustworthy adoption [21,25,37]. Therefore, the complex nature of clinical decisions is said to call for the transparent traceability of the logic leading to output and how clinicians interact with what they understand from explanations, thus requiring guidance as to what constitutes satisfactory explanations of decisions and how such resources should be documented [21,22,26,32]. In the United States, for products that do not fall under the scope of FDA regulation, reporting guidelines (TRIPOD [Transparent Reporting of a Multivariable Prediction Model for Individual Prognosis or Diagnosis], CONSORT [Consolidated Standards of Reporting Trials], IEEE, or STROBE [Strengthening the Reporting of Observational Studies in Epidemiology]) that support the transparent documentation of software development and validation exist but are inconsistently adopted and do not show evidence of driving analysis and documentation throughout all areas of product development, evaluation, deployment, and postdeployment [25,28,29,39].

## Discussion

### Principal Findings

With an understanding of the current state and challenges of AI- and ML-based documentation, addressing present barriers, including those identified in this scoping review as involving bias, accountability, governance, and explainability, is required to enhance documentation efforts and promote the translation of MMS. Recommendations identified in the literature call for a proactive evaluation of MMS, multidisciplinary collaboration, adherence to investigation and validation protocols, transparency and traceability requirements, and guiding standards and frameworks that enable innovation across translational aspects and support MMS throughout the software life cycle.

Documentation serving to proactively outline and guide translational processes and encourage multidisciplinary collaboration is recommended to support system development throughout the AI life cycle to promote patient safety, support appropriate clinical use cases, and ensure that essential testing and validation processes are completed before deployment [22-24,28]. Proactively accounting for translation reflects ex ante (as opposed to ex post) regulation that is “pre-emptive of foreseeable risks and has a more open and participatory character” [25,32]. To help facilitate the proactive evaluation of MMS, researchers such as Wiens et al [35] and Allen et al [24] suggest the need for a road map for deploying AI- and ML-based tools that consist of a stepwise framework that introduces methods and tools for development, testing, validation, and monitoring from the beginning of production (problem identification and idea formulation) to the end (widespread deployment and maintenance).

Such a road map is said to be enhanced by multidisciplinary collaboration, promoting robust partnerships among stakeholders and project teams through iterative and joint approaches such as participatory design [25,35]. Within many of the proposed and available road maps, frameworks, and recommendations reviewed in the scoping review, the engagement of stakeholders is encouraged to occur early in the process to ensure the development of an optimal solution; this includes helping to determine clinical relevance, identifying appropriate data and collaborators, considering ethical implications and engaging with ethicists, rigorous evaluations and reporting of predictions and model code, organizing clinical trials and safety monitoring of strategies, and market deployment approaches, all of which ought to be documented and transparent to the interdisciplinary team throughout the product’s life cycle [24,35,40].

Communicating evidence of adherence to investigation and validation protocols was said to support MMS implementation, as it provides an objective measure of testing efforts and is especially important for instances of uncertainty and conflict between AI and clinicians to bolster traceability for liability and legal obligations and mitigate ethical concerns [13,21,25,26]. Providers can use the documentation of adherence to such protocols in practice, as provider explanations to end users are said to mitigate concerns and threats of overreliance on MMS through the ability to communicate and document the training for decision logic, system output, and role definition in the final decisions made for patients [13,21]. Leveraging the evidence of investigation and validation is critical for ensuring representativeness and appropriate clinical application to minimize the consequence of patient harm and inequitable model performance. In addition, by using transparent documentation, Ploug and Holm [36] also propose “contestability by design,” an approach that focuses on system development and optimization and provides “design principles for algorithmic systems that will enable professionals and expert users to challenge the reasoning of these systems in an ongoing process” in a way that does not come at the cost of system performance and supports the introduction of a minimal set of criteria to serve as a practical guideline.

Documenting such efforts fulfills the recommendations for promoting transparency and traceability requirements suggested to support the explainability of MMS. Explanations, referred to as a “cardinal responsibility of medical practitioners,” function as an additional safeguard to ensure the reliability of a system’s reasoning process, whereas “trust” in clinical decisions correlates with the construct of explainability at all levels of expertise [21,27]. The complexity of reasoning underlying explanations highlights the need to address the varying definitions of explainability (and interpretability) and the extent to which they are required to effectively support MMS and manage the inconsistency and vagueness surrounding bias and the implications of equity and patient safety [21,27]. These requirements span beyond the scope of 1 team within a project, making explainability a necessity to be adopted and prioritized from a multidisciplinary perspective in a way that promotes knowledge continuity across disciplines; provides relevant explanations of MMS logic for both technical and translational purposes; and provides evidence of MMS traceability [13,21,22,24,27,39].

Investigation and validation protocols and transparency and traceability requirements were also recommended to be aided by guiding standards and frameworks, from reporting guidelines developed by research groups to regulatory protocols published by governance bodies [13,21,23,24,29,37]. Although a consensus for universal best practices was not recognized in the literature, the importance of leveraging guiding standards and frameworks was consistently identified, from proposed reporting guidelines published by research teams to legislation defined by governing bodies [22,23]. Available and proposed standards, frameworks, and governance structures to promote ethical and explainable AI- and ML-based MMS documentation and translation mentioned in this scoping review’s literature were recorded. They are available in Multimedia Appendix 5 (ethics) and Multimedia Appendix 6 (explainability).

Integrating recommendations related to proactive evaluation; multidisciplinary collaboration; and adherence to investigation and validation protocols, transparency and traceability requirements, and guiding standards and frameworks are expected to enhance documentation efforts by increasing the transparent reporting of scientific evidence, promoting knowledge continuity across disciplines, providing guidance throughout the AI life cycle, and improving usability in clinical practice while also addressing the implementation gap.

### Limitations

Limitations of this study include the restriction of the search to only 1 database, PubMed, to conduct the scoping review. This limitation likely restricted the available and proposed governance standards, guidelines, and frameworks. Notably, additional resources found in our previous studies were not explicitly mentioned in the literature, for example, Google’s Model Card for model reporting [45], Model Facts Labels [46], DECIDE-AI (Developmental and Exploratory Clinical Investigations of Decision support systems driven by Artificial Intelligence) [47], and AI Factsheets [48]. Therefore, continuing this investigation as other resources are available (eg, Coalition for Health AI guardrails [49] and National Institute of Standards and Technology recommendations [50]) is critical. In addition, owing to the exploratory nature of scoping reviews, this study aimed to assess the available academic literature, organize findings within themes, and highlight present gaps regarding AI- and ML-based MMS documentation and translation practices rather than explore a more defined research question. This study also is limited to scoping the concerns in existing literature; the extent to which these concerns may be incomplete, or misguided, is not within our scope, for example around the question of when AI and ML may be not just inappropriate but illegitimate [51], or the danger that practitioners mistakenly think that explainable models reflect causality in the world [52-55] and therefore base their trust and decision-making on a fundamental error.

### Implications for Practice and Future Development

This study identifies a need for proactive evaluation, standards, frameworks, and transparency and traceability requirements to enhance documentation efforts and promote the translation of MMS. However, how might practitioners achieve these goals? What steps can be taken to improve documentation efforts and promote transparency and traceability, and how might they be implemented into practice?

As indicated by the findings in this scoping review, although there are existing standards, guidelines, frameworks, and governance structures to guide the documentation of AI- and ML-based MMS, there is acknowledgment that these are insufficient and that additional resources are needed to provide appropriate guidance related to ethics and explainability, promote safety and efficacy, provide support throughout the AI life cycle, and reduce present barriers to translation. The available but fragmented and phase-specific resources create an opportunity either to streamline and merge complementary standards, guidelines, frameworks, and governance structures or to encourage the development of new resources. Recommendations highlighted from the literature may help inform the development of such new resources to operationalize the theoretical into actionable tools that answer questions such as, How do I know if the implementation of an MMS tool is the right solution? What agreed-upon principles can be leveraged to analyze data, build tools, and guide implementation? What level of evidence and types of study designs will be expected to assess model effectiveness in terms of validity, acceptability, fairness, equity, transparency, and health impact? How can external validation of models be facilitated? What processes are required to effectively document and navigate the regulatory and quality assurance pathway? and What constitutes effective postdeployment monitoring?

The proactive and standardized evaluation and documentation of MMS through a multidisciplinary collaboration of accountable contributors may contribute to the operationalization of guidelines. By promoting enterprise adherence to investigation and validation protocols, clinician judgment (eg, model output thresholding), and traceability requirements, such an approach aligns evidence-based best practices. Conceptually, the central testing, documentation, and multistakeholder coordination applied to patient’s longitudinal electronic health record is applied to the AI model throughout its life cycle.

Such a unified model document would represent a framework for easy discovery of the critical scientific, ethical, and regulatory requirements of an AI- and ML-based MMS while allowing for future expandability or customization to fit the needs of each specialty area and possible future requirements. This unified model document could be shared across all levels of model development, from the ideate phase to postdeployment monitoring. Ideally, the unified model document would capture the fundamental features of any AI- and ML-based MMS while guiding accountable stakeholders on how to address issues. For example, if an MMS developer answers a question about explainability as a “black box,” it could provide some basic recommendations for expanding explainability. The document could be created and translated to a standard markup language such as XML or JSON. This would allow it to have a formal structure for the documentation but allow for consumption by others for implementation (eg, Epic and Cerner). Moreover, large institutions that routinely use these tools in clinical practice (eg, health care centers) could adopt a risk-based approach, creating a centralized team of experts to standardize the categorization of all AI- and ML-based MMS tools and determine the type of control measures, evidence, and overall rigor required for their safe and effective development, deployment, and monitoring.

In addition to the unified model document, a possible direction of the industry is to follow others who develop tools and architectures that follow a DevOps framework for ML called Machine Learning Operations. The first step could be at the national level, through regulatory bodies (the Office of the National Coordinator for Health Information Technology, FDA, IEEE, etc), medical academies (National Academy of Medicine, American Medical Informatics Association, American Medical Association, American College of Obstetricians and Gynecologists, American Academy of Family Physicians, American College of Surgeons, etc), or industry and academic collaboration (Google, Microsoft, Amazon, Epic, etc) to develop a set of practice standards, similar to other regulations such as SaMD, which are adopted across the AI- and ML-based MMS industry and require specific documentation and disclosure of MMS technologies that create consistency throughout development to the release to the public. In addition, organizations could create enterprise AI- and ML-based MMS translational boards comprising various experts in the fields of AI and ML, including data science or engineering, IT, ethics, electronic health records, nursing or clinical or translational or pharmacy informatics, and clinical expertise. Similar to an institutional review board found at any research institution, the AI and ML translational board would help address gaps in ethics or bias, explainability, and efficacy and reduce the translational barriers. Finally, institutions and medical colleges could require training and regular refresher courses for clinicians about AI- and ML-based MMS tools that would follow up-to-date standards of practice with many other medical devices (Clinical Laboratory Improvement Amendments, Long-Acting Reversible Contraception training, Basic Life Support and Advanced Cardiac Life Support, etc).

As health centers are venturing into the development of SaMD’s internal deployment and commercialization, an enterprise-wide system to enable guided development without stifling innovation may prove to be valuable. To achieve this goal, the transparency and traceability of documentation should align with requirements for FDA submission and Centers for Medicare and Medicaid Services and Joint Commission compliance while leveraging the best practices of technical stakeholders and internal oversight. Such a coordinated effort could be implemented in practice by deploying an enterprise quality management system for AI development, translation, and continuous monitoring, such that internal and external evaluation reports become standardized artifacts for inclusion in MMS documentation. Stakeholders across the AI life cycle would contribute to this work, each considered accountable for subject matter expertise and their action in the quality management system. Examples of stakeholders may include data scientists, informaticists, software engineers, translational and implementation scientists, educators, and providers. Each stakeholder can enhance transparency by increasing their awareness about risk in their domain, and dependencies associated with their action and documentation. For example, a data scientist’s selection of clinical features will have substantial implications for the application of the model. This information, if transparently documented, will serve clinical stakeholders, informaticians, and translational scientists when discussing model performance, tuning, and deployment. Further down the AI translation process, the implementation scientist’s workflow evaluation and documentation of model insertion points will be leveraged to facilitate discussions between data scientists and clinicians to appropriately threshold and present model output information based on the documented considerations of model risk and performance. Promoting traceability and transparency for national and international reportable standards is in process. For example, there are ongoing efforts to draw consensus around current best practices and recommendations for standardized AI evaluation and governance in health care; examples include the Coalition for Health AI [49], Health AI Partnership [56], and WHO and International Telecommunications Union Focus Group on Artificial Intelligence for Health [10]. Meanwhile, the National Academy of Medicine is developing an AI Code of Conduct [57], which may be an opportunity to define each stakeholder category and clarify the determination of distributed accountability and responsibility. Academic health centers have begun forming governance bodies that enforce evaluation and checkpoints, with accompanying AI- and ML-based MMS tool artifacts. If aggregated across the AI life cycle, such artifacts could be the foundation for MMS documentation [58] and evolve with regulations. In their development, oversight bodies may leverage published AI translation evaluation frameworks and join or learn from ongoing studies in this area that is actively developing detailed guidelines.

### Conclusions

To know whether promises about how the adoption of AI- and ML-based MMS tools has the power to revolutionize health care are valid, and to then achieve such benefits, requires strategic translation that prioritizes ethical considerations, the ability to provide explanations that transparently communicates how a decision is reached, and disclosures on the intended use that are accessible to multidisciplinary perspectives across experts and nonexperts. The ability of MMS to deliver on expectations depends on resolving translation barriers, including those related to bias, accountability, governance, and explainability. Our findings suggest that aligning translational and regulatory science through strategic documentation developed to promote proactive evaluation, multidisciplinary collaboration, investigation and validation protocols, transparency and traceability requirements, and guiding standards and frameworks will support the translation and adoption of MMS. Further, we propose that leveraging such transparent documentation processes through a quality management system may support enterprise coordination toward the development of an SaMD regulatory framework.

## Appendix

Multimedia Appendix 1 Key concepts and search limitations used to generate the scoping review query, including the keywords and Medical Subject Headings (MeSH) terms and date and journal constraints.

Multimedia Appendix 2 Data extraction template developed on Covidence to standardize information extracted from studies to satisfy scoping review objectives.

Multimedia Appendix 3 PRISMA-ScR (Preferred Reporting Items for Systematic Reviews and Meta-Analyses extension for Scoping Reviews) checklist.

Multimedia Appendix 4 Overview of publications included in the scoping review (N=21).

Multimedia Appendix 5 Available and proposed standards, guidelines, frameworks, and governance structures to promote ethical artificial intelligence– and machine learning–based medical modeling software documentation and translation mentioned throughout literature.

Multimedia Appendix 6 Available and proposed standards, legislation, organizations, guidelines, frameworks, and governance bodies to promote explainable artificial intelligence– and machine learning–based medical modeling software tool documentation and translation mentioned throughout literature.

## Abbreviations

AI: artificial intelligence
CDS: clinical decision support
CONSORT: Consolidated Standards of Reporting Trials
DECIDE-AI: Developmental and Exploratory Clinical Investigations of Decision support systems driven by Artificial Intelligence
FDA: Food and Drug Administration
IEEE: Institute of Electrical and Electronics Engineers
ML: machine learning
MMS: medical modeling software
PRISMA-ScR: Preferred Reporting Items for Systematic Reviews and Meta-Analyses extension for Scoping Reviews
SaMD: software as a medical device
STROBE: Strengthening the Reporting of Observational Studies in Epidemiology
TRIPOD: Transparent Reporting of a Multivariable Prediction Model for Individual Prognosis or Diagnosis
WHO: World Health Organization

## Data Availability

All data generated or analyzed during this study are included in this published paper and the Multimedia Appendices 1-6.
