# Supplementary material for: The Role of Artificial Intelligence Model Documentation in Translational Science: Scoping Review
Source: Interact J Med Res. 2023 Jul 14;12:e45903. doi: 10.2196/45903 (PMC10382950; doi:10.2196/45903)
Supplement: Multimedia Appendix 1 [file ijmr_v12i1e45903_app1.pdf]

Key Concept	Keywords	MeSH Terms	Key Concept Search Query
Artificial Intelligence / Machine Learning	Artificial intelligence, machine learning	"Artificial Intelligence"[Mesh] OR "Machine Learning"[Mesh] OR "Decision Support Systems, Clinical"[Mesh]	"artificial intelligence"[Text Word] OR "machine learning"[Text Word] OR "Artificial Intelligence"[Mesh] OR "Machine Learning"[Mesh] OR "Decision Support Systems, Clinical"[Mesh]
Ethical Considerations	Explainability, interpretability, governance, documentation, translation, translational informatics	"Clinical Governance"[Mesh] OR "Documentation"[Mesh] OR "Standard of Care"[Mesh]	explainability[Text Word] OR interpretability[Text Word] OR governance[Text Word] OR documentation[Text Word] OR translat*[Text Word] OR “translational informatics”[Text Word] OR "Clinical Governance"[Mesh] OR "Documentation"[Mesh] OR "Standard of Care"[Mesh]
Explainability	ethical consideration*, ethic(s), bias(es), algorithmic bias	"Ethical Analysis"[Mesh] OR "Ethics, Clinical"[Mesh] OR "Selection Bias"[Mesh]	ethical consideration*[Text Word] OR ethic*[Text Word] OR bias*[Text Word] OR "algorithm bias"[Text Word] OR "Ethical Analysis"[Mesh] OR "Ethics, Clinical"[Mesh] OR "Selection Bias"[Mesh]

	Search Limitation	Query
Database	PubMed	
Date	Publication after 2015	2015/01/01:2022/2/1[Date - Publication]
Journal	Journals identified to be relevant to the study objectives as defined by Jane (cite Jane)	(Nature medicine"[Journal] OR "J Am Med Inform Assoc."[Journal] OR "PLoS One"[jour] OR “BMJ Open”[Journal] OR "Bull World Health Organ"[jour] OR “IEEE Trans Vis Comput Graph”[Journal] OR “Bioethics”[Journal] OR “J Oral Biol Craniofac Res”[Journal] OR “BMC Med Inform Decis Mak”[Journal] OR “JMIR Med Inform”[Journal] OR “J Med Internet Res”[Journal] OR “Stud Health Technol Inform”[Journal] OR “J Am Coll Radiol”[Journal] OR “Yearb Med Inform”[Journal] OR “Artif Intell Med”[Journal] OR “Clin Radiol”[Journal] OR “Comput Biol Med”[Journal] OR “Comput Methods Programs Biomed”[Journal] OR ”Int J Med Inform”[Journal] OR “Sci Eng Ethics”[Journal] OR “Implement Sci”[Journal] OR “J Med Ethics”[Journal] OR “Comput Inform Nurs”[Journal])

**Full PubMed Search Query**:
("Artificial Intelligence"[Mesh] OR "Machine Learning"[Mesh] OR "Decision Support Systems, Clinical"[Mesh] OR "artificial intelligence"[Text Word] OR "machine learning"[Text Word]) **AND** ("Clinical Governance"[Mesh] OR "Documentation"[Mesh] OR "Standard of Care"[Mesh] OR explainability[Text Word] OR interpretability[Text Word] OR governance[Text Word] OR documentation[Text Word] OR translat*[Text Word] OR “translational informatics”[Text Word]) **AND** ("Ethical Analysis"[Mesh] OR "Ethics, Clinical"[Mesh] OR "Selection Bias"[Mesh] OR ethical consideration*[Text Word] OR ethic*[Text Word] OR bias*[Text Word] OR "algorithm bias"[Text Word]) **AND** (2015/01/01:2022/2/1[Date - Publication]) **AND** (Nature medicine"[Journal] OR "J Am Med Inform Assoc."[Journal] OR "PLoS One"[jour] OR “BMJ Open”[Journal] OR "Bull World Health Organ"[jour] OR “IEEE Trans Vis Comput Graph”[Journal] OR “Bioethics”[Journal] OR “J Oral Biol Craniofac Res”[Journal] OR “BMC Med Inform Decis Mak”[Journal] OR “JMIR Med Inform”[Journal] OR “J Med Internet Res”[Journal] OR “Stud Health Technol Inform”[Journal] OR “J Am Coll Radiol”[Journal] OR “Yearb Med Inform”[Journal] OR “Artif Intell Med”[Journal] OR “Clin Radiol”[Journal] OR “Comput Biol Med”[Journal] OR “Comput Methods Programs Biomed”[Journal] OR ”Int J Med Inform”[Journal] OR “Sci Eng Ethics”[Journal] OR “Implement Sci”[Journal] OR “J Med Ethics”[Journal] OR “Comput Inform Nurs”[Journal])
