# Supplementary material for: The Role of Artificial Intelligence Model Documentation in Translational Science: Scoping Review
Source: Interact J Med Res. 2023 Jul 14;12:e45903. doi: 10.2196/45903 (PMC10382950; doi:10.2196/45903)
Supplement: Multimedia Appendix 2 [file ijmr_v12i1e45903_app2.pdf]

**Data Extraction Template (developed in Covidence)**	
**General Information of Publication**	Title
	Authors
	Year
	Country
**Publication Characteristics**	Study design
	Topics included (AI/ML, Documentation, Governance, Ethical considerations, Explainability, and/or Translation)
	Objective of publication
	Key words
**Research Objective Specific Information**	Present challenges in AI/ML
	Ethical considerations (Identified gaps and barriers / Recommendations)
	Explainability (Identified gaps and barriers / Recommendations)
	Standards, regulations, best practices, or governance strategies mentioned
	Overall recommendations from paper
	Relevant quotes
	Additional relevant information
